# Identification of Plant Peroxidases Catalyzing the Degradation of Fluorinated Aromatics Using a Peroxidase Library Approach

**DOI:** 10.1002/elsc.202400054

**Published:** 2024-09-17

**Authors:** Ashton Ware, Sally Hess, David Gligor, Sierra Numer, Jack Gregory, Carson Farmer, Gregory M. Raner, Hector E. Medina

**Affiliations:** ^1^ Department of Biology and Chemistry Liberty University Lynchburg Virginia USA; ^2^ School of Engineering Liberty University Lynchburg Virginia USA

**Keywords:** biodegradation, bioreactor, catalysis, peroxidase, PFAS

## Abstract

In this work, the degradation of mono‐ and polyfluorinated phenolic compounds was demonstrated by a series of crude plant peroxidases, including horseradish root (HRP) and six members of the *Cucurbita* genus. Highly active samples were identified using a library screening approach in which more than 50 crude plant samples were initially evaluated for defluorination activity toward 4‐fluorophenol. The highest concentrations were observed in the HRP, pumpkin skin (PKS), and butternut squash skin (BNS), which consistently gave the highest intrinsic rates of decomposition for all the substrates tested. Although HRP exhibited a significant decrease in activity with increased fluorination of the phenolic substrate, PKS showed only minor reductions. Furthermore, in silico studies indicated that the active site of HRP poorly accommodates the steric bulk of additional fluorines, causing the substrate to dock farther from the catalytic heme and thus slowing the catalysis rate. We propose that the PKS active site might be larger, allowing closer access to the perfluorinated substrate, and therefore maintaining higher activity compared to the HRP enzyme. However, detailed kinetic characterization studies of the peroxidases are recommended. Conclusively, the high catalytic activity of PKS and its high yield per gram of tissue make it an excellent candidate for developing environmentally friendly biocatalytic methods for degrading fluorinated aromatics. Finally, the success of the library approach in identifying highly active samples for polyfluorinated aromatic compound (PFAC) degradation suggests the method may find utility in the quest for other advanced catalysts for PFAS degradation.

**Abbreviations**
4FP: 4‐fluorophenol
BNS: butternut squash skin
BSA: bovine serum albumin
DFP: 3,4‐difluorophenol
HPLC: high‐performance liquid chromatography
HRP: horseradish root
PFAC: polyfluorinated aromatic compound
PFAS: per‐ and polyfluoroalkyl substance
PFP: 2,3,4,5,6‐pentafluorophenol
PKS: pumpkin skin
TFP: 3,4,5‐trifluorophenol

## 1 Introduction

Per‐ and polyfluoroalkyl substances (PFASs) are surfactant‐like, anthropogenic compounds characterized by containing at least one moiety –C*_n_* F_2_ *_n_*. PFAS, commonly referred to as “forever chemicals”, is a family of more than 14k—although this list is growing—molecules of between ∼50 and ∼3500 Da (with an average of ∼400 Da), exhibiting both aliphatic and aromatic structures. The physical and chemical properties of the C─F bond have led to the enhanced development of numerous fluorinated hydrocarbons in a large variety of applications, including pharmaceutical and agrochemical agents [1, 2, 3, 4, 5, 6]. Consequently, even though organofluorines are not commonly synthesized biologically, their environmental presence is on the rise in response to the substantial increase in their industrial production. For example, a large number of fluorinated drugs and their metabolites have been detected in ground water sources throughout the world, including fluoxetine, sitagliptin, and levofloxacin, to name a few [7, 8, 9]. Furthermore, many of these fluorinated compounds have been shown to have potentially adverse effects on the environment and/or human health [10, 11]. For example, Panieri et al. prepared a comprehensive review [12] of in vitro studies suggesting potential health risks associated with PFAS exposure, including oxidative stress [13, 14], apoptotic cell death [15, 16], increased intracellular triglyceride accumulation [17], decreased bile acid metabolism [18], inhibition of drug metabolism [19], and perturbations in Ca^2+^ concentrations in neurons [20]. Furthermore, measurable quantities of PFAS have been detected in human blood, serum, and urine, underscoring the significance of the potential health effects previously described [21, 22, 23, 24, 25, 26, 27, 28]. Analysis of effluents from wastewater treatment plants suggests that current methods employed for the removal of these toxicants are somewhat ineffective, which may explain the presence of these compounds in human tissue, thus highlighting the need for the development of more efficient catalysts for their degradation.

**Summary**

- The solution to the per‐ and polyfluoroalkyl substance (PFAS) problem worldwide has been estimated at near $200T, which is about double the current global GDP. Not only is important to remove PFAS from the various environmental matrices—for example, water, soil, and air—but also important is the need to completely destroy these persistent anthropogenic molecules.
- Current destruction solutions, such as incineration, are energy intensive and do not guarantee destruction. Bioremediation methods might be the most promising routes to completely destroy these contaminants without impacting too much the economy, especially in disadvantaged regions of the world.
- The implementation of low‐cost peroxidases, such as pumpkin skin (PKS) from our study, in combination with easy‐to‐make and low‐cost photocatalytic enhanced surface area structures could lead to the design of low‐cost bioreactors capable of efficiently destroy PFAS molecules for once and for all.

In recognition of this problem, Wang and Liu reviewed a variety of metalloenzyme catalysts capable of promoting C–F bond cleavage [29]. Among these, potential catalysts were tyrosine hydroxylases, cytochrome P450 monooxygenases, heme‐thiolate peroxygenases, bifunctional dehaloperoxidases, and non‐heme oxygenases. A notable omission in this review was the classical plant peroxidase from horseradish root (HRP), which has been shown to catalyze 4‐fluorophenol (4FP) defluorination [30]. It was noted in this study that inactivation of the enzyme was observed, which could be a limitation for its use. Yet, it was also highlighted that the timely and proper addition of bovine serum albumin (BSA) was able to spare the enzyme from inactivation. Although HRP is only one of thousands of plant peroxidases in nature, it is the one most prominently featured in biotechnological applications, due to its high activity, abundance in the HRP, and its availability via recombinant expression systems. The nearly exclusive use of this specific enzyme to assess the suitability of plant peroxidases for biotechnological applications has the potential to overlook members of this family possessing more desirable or novel catalytic properties. In the degradation of fluorinated phenols, for example, other peroxidase family members may possess similar or better catalytic properties. Furthermore, the availability and cost of recombinant HRP may also be a limiting factor for use in water treatment in developing nations. Thus, identifying more abundant natural sources from alternate biomaterial is a desirable objective also addressed in this research. Therefore, the current study was developed as a systematic approach to identify peroxidases from various plant sources with high catalytic activity in the oxidation of fluorinated phenolic compounds, or to identify family members with novel chemical behavior toward these fluorinated compounds. A crude peroxidase library has been constructed from more than 100 different plant sources using a standardized isolation protocol. A high‐performance liquid chromatography (HPLC) screening protocol was developed to assess metabolic activities within the library, and highly active samples were purified for kinetic analysis for direct comparison with HRP in the metabolism of four different fluorinated phenolic compounds: 4FP, 3,4‐difluorophenol (DFP), 3,4,5‐trifluorophenol (TFP), and 2,3,4,5,6‐pentafluorophenol (PFP). Several peroxidases were identified with very high catalytic efficiency toward these substrates, including an enzyme from pumpkin skin (PKS), which routinely yielded more than 10x as much peroxidase/g of tissue than nearly any other sample examined, validating the idea that this library approach can be an effective tool in the development of more efficient or novel catalysts in biotechnology research. It should be noted that strategies for the degradation of fluorinated aliphatic compounds may be very different than those that are effective with fluorinated aromatics, given the higher bond energies associated with the aliphatic compounds. Nevertheless, a robust peroxidase library of the type described here may also be applied for the development of novel methodologies in PFAS degradation across multiple classes.

## 2 Materials and Methods

### 2.1 Crude Sample Preparation

Raw material was collected for each plant type and weighed prior to the homogenization process. A 20 mM potassium phosphate buffer (pH 6.5) was added to each sample in a 2:1 volume‐to‐mass ratio. A mortar and pestle were utilized to crush the material thoroughly while submerged in buffer at 23°C. The material was then centrifuged for 15 min at 10,000 × *g* and 4°C. Aliquots of the resulting supernatant were acquired and diluted for each of the activity measurements. Three dilutions (1:10, 1:100, and 1:1000) were made for each species and all sample dilutions were made using 20 mM phosphate buffer (pH 6.5) as the diluent. Aliquots of 1.0 mL of each of the samples generated were stored at −20°C as a part of the crude peroxidase library. For this research, a total of 80 samples were evaluated with the 44 most active shown in Table 1.

**TABLE 1:** Identity of the most active crude peroxidase samples identified for the study along with their abbreviations.

Sample	Abbreviation	Sample	Abbreviation
Butternut squash skin	BNS	Horseradish root	HRP
Pumpkin skin	PKS	Brussel sprout	BRUS
Kudzu leaf	KUD	Catnip leaf	CAT
Zucchini skin	ZUS	Yellow squash skin	YS
Radish stem	RAS	Sweet potato skin	SWP
Radish skin	RRS	Carrot skin	CARS
Russett potato skin	RUS	Broccoli stem	BRS
Broccoli head	BRH	Zucchini seed	ZS
Green pea	GRP	Green bean skin	GBS
Mint leaf	MIN	Green bean pod	GBP
Red lettuce leaf	RLL	Radish leaf	RAL
Hydrangea leaf	HYD	Red chard stalk	RED
Ginger flesh	GIF	Apple seed	APS
Ginger skin	GIN	Purple heart leaf	PUR
Cauliflower	CAU	Bok choy	BOK
Red chard leaf	REL	Pothos leaf	POL
Jalepeno seed	JAL	Clover leaf	CLL
Pumpkin seed	PSS	Clover flower	CLF
Pumpkin flesh	PKF	Japanese pagoda	JAP
Dandelion flower	DNF	Dandelion leaf	DWL
Red lettuce skin	RLS	Red lettuce leaf	RLL
Pumpkin pulp	PKP		

### 2.2 Guaiacol Assay

A standard guaiacol assay mix containing 5.0 mM guaiacol, 5.0 mM H_2_O_2_, and 20 mM phosphate buffer (pH 6.5) was prepared and used for all screening reactions involving crude peroxidase samples. This solution was made fresh each day. The screening reactions consisted of 20 µL of each sample type in a vial containing 980 µL of the assay mixture at 23°C. A spectrophotometric kinetics assay was then performed measuring absorbance change for 1 min at 470 nm, measured every 10 s, at 23°C. Activities were calculated based on the molar absorptivity of tetraguaiacol (26.6 mM^−1^ cm^−1^) and recorded in Units (U), where 1 U is equal to µmoles guaiacol formed per min. Serial dilutions of 1:10, 1:100, and 1:000 were made for each sample as deemed necessary based on the magnitude of their crude activities. Experiments were performed in triplicate for each of the samples, and the values given are the average of the three. Standard deviations were calculated and were all within 10% of the measured average value for each experiment.

### 2.3 Fluorophenol Assay

Library screening for 4FP oxidation was carried out using an HPLC assay developed to quantify the primary product of the reaction benzoquinone. For these reactions, 20 µL of each crude sample was added to 980 µL of a 4FP assay mix containing 5.0 mM 4FP, 5.0 mM H_2_O_2_, and 20 mM phosphate buffer (pH 6.5). The reaction was carried out for 5 min at 30°C and then quenched with ice‐cold 2:1 mixture of methanol:HCl (0.1 M) and placed on ice for an additional 10 min. The samples were then centrifuged at 10,000 × *g* for 5 min at 23°C. The supernatant was transferred to HPLC vials for analysis using a 50 mm RP C18 HPLC column (5 µm) connected to an Agilent Integrity1260 automated HPLC system with a quaternary mixing system and in‐line degassing coupled to diode array absorption detection module. An isocratic mobile phase consisting of 40% acetonitrile and 60% H_2_O, both containing 0.1% TFA (trifluoroacetic acid), was used at a flow rate of 1.2 mL min^−1^. The product, benzoquinone, was detected using absorption detection at 254 nm, and a standard curve was generated and used to quantify product formation for each crude sample. Under these conditions, the benzoquinone had an elution time of approximately 1.9 min.

### 2.4 Heme Determination

Total heme content for the six *Cucurbita* crude samples along with that of the HRP was determined using HPLC. A 50 µL sample of the crude protein was treated with 500 µL of a 1:1 mixture of acetonitrile and H_2_O containing 0.1%TFA. After a 10 min incubation on ice, 20 µL of the sample was analyzed by HPLC using an RP C18 column (5 × 150 mm) with a mobile phase of 1:1 acetonitrile/H_2_O and a flow rate of 1.0 mL min^−1^, with detection at 398 nm. Under these conditions, the released heme cofactor had a retention time of 6.0 min. A standard curve was prepared using known concentrations of a commercial sample of HRP (Sigma–Aldrich), where the concentrations of the standard solutions were determined using the molar absorptivity of HRP (1.02 × 10^5^ M^−1^ cm^−1^ at 403 nm) and this curve was linear up to 50 µM HRP.

### 2.5 Assays for PFP, TFP, DFP, and 4FP Degradation

The screening reactions described above were performed by looking at benzoquinone product formation; however, distinct products in the tri‐ and penta‐fluorophenol reactions were not observed. Therefore, to compare directly the four compounds, the decomposition of substrate was monitored in the reactions. All reactions consisted of 1.0 mM substrate and 5.0 mM H_2_O_2_ in 50 mM phosphate buffer (pH 6.5) and the corresponding concentrations of each enzyme in a total volume of 0.500 µL. Enzyme concentrations were all normalized (0.06 mM) based on heme content, and reactions were carried out for 2.0 and 10.0 min at 23°C. Reactions were quenched by the addition of 200 µL methanol and 100 µL of 1.0 M HCL. For HPLC analysis, 20 µL of each sample was injected onto a 250 × 4.6 mm (5 mm) BDS Hypersil C18 column (Thermo) at a flow rate of 1.2 mL min^−1^. Mobile phase compositions and detection wavelengths were as follows: (1) for PFP, 50% A and 50% B with detection at 265 nm; (2) for TFP, 50% A and 50% B with detection at 230 nm; and (3) for DFP and 4FP, 60% A and 40% B with detection at 275 nm; where solvent A was water with 0.1% TFA and B was 100% acetonitrile containing 0.1% TFA.

### 2.6 Docking Studies

For docking, the HRP crystal structure from Berglund et al., PDB: 1H5A, [31] was used for the study. The structure was cleaned and repaired in ChimeraX [32] and the ADFR Suite [33]. The ligands for docking were prepared with the ADFR Suite. Docking was performed with AutoDock Vina 1.2.5 [34]. The center of the docking position was set to *x* = 2.3 Å, *y* = 2.58 Å, and *z* = 11.57 Å with box sides of 7.5 Å. The exhaustiveness was set to 100, and all other settings were as prescribed by default in those available packages.

## 3 Results and Discussion

### 3.1 Library Screening for Guaiacol and 4FP Oxidation

The initial crude sample preparations resulted in a very wide range of guaiacol oxidation activities, and as expected, more active samples produced signals outside of the linear range. Table 1 lists the most active plant samples utilized in this study along with the abbreviations used.

**FIGURE 1:**
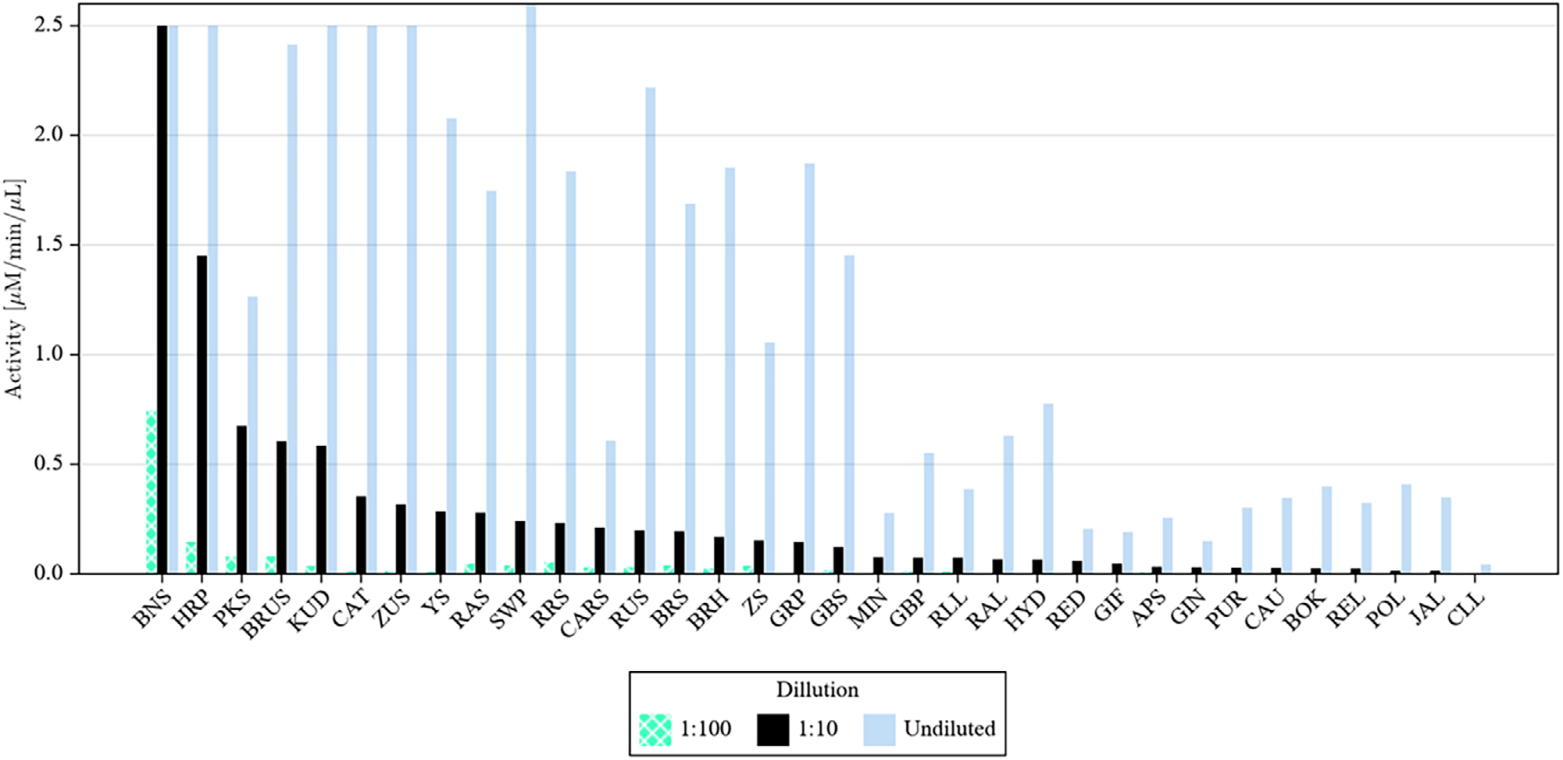
Graphical representation of guaiacol oxidation data acquired for all 50 crude peroxidase samples along with 1:10 and 1:100 dilutions. The 1:10 data is highlighted in the black solid bars as this gives a very good visual for the overall distribution of activities. A minimum of three values for each sample were acquired and triplicate values all agreed to within 10% of the average value for each sample.

A serial dilution approach was adopted to differentiate the more highly active samples. The resulting activity profiles are displayed in Figure 1. The middle black bars within each set represent the 1:10 dilution of the crude peroxidase samples and give the best overall perspective of relative activities. The only undistinguished samples were butternut squash skin (BNS) and HRP, since both gave saturating activities in the absorption assay that was used. Here, a 1:100 dilution was used to rank their overall activities. Based on these data, the BNS crude extract showed the highest overall activity, outperforming the HRP by a factor of more than 10‐fold. It should be noted that all the skin samples did show some variability from distinct preparation trials (up to 50%), presumably due to the peeling process itself. Since all values were normalized based on the total mass of sample, deeper peeling would retain a higher mass of the inactive pulp, thus reducing the relative activity of the material. However, even with this variability, the crude butternut squash and PKSs, along with HRP, universally displayed the highest relative activities. Several other squash family members also displayed high relative peroxidase activities, including yellow squash, buttercup squash, acorn squash, zucchini, and spaghetti squash. It is noteworthy that several prior studies addressing BTS focused primarily on the flesh rather than skin, which is interesting since in our study, only the skin demonstrated significant activity. The current data suggest that the *Cucurbita* family is a rich source of peroxidase enzymes, particularly the peeling from these sources.

**FIGURE 2:**
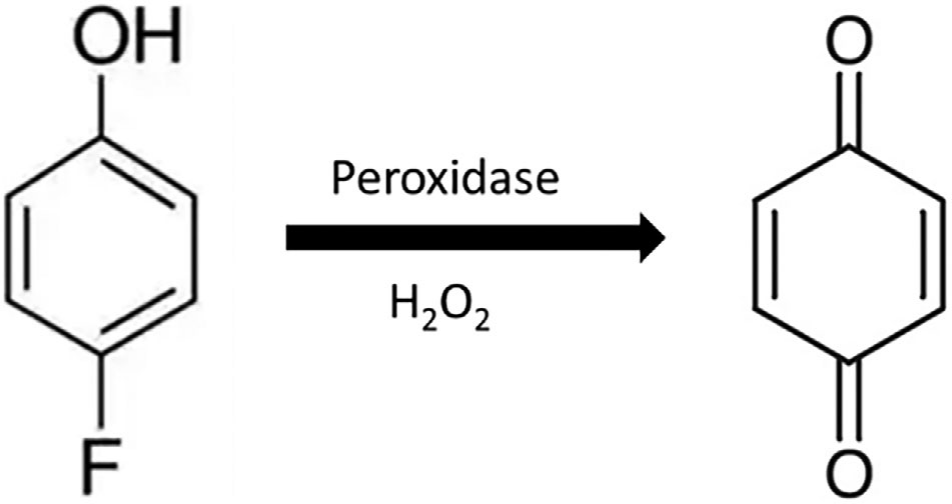
Reaction used to monitor fluorophenol degradation by crude peroxidase library.

**FIGURE 3:**
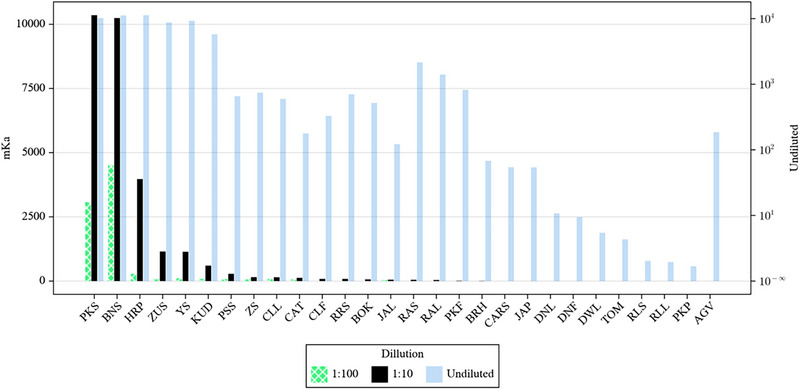
Graphical representation of 4‐fluorophenol oxidation data acquired for crude peroxidase samples along with 1:10 and 1:100 dilutions. The data are shown on a log scale. The 1:10 data is shown in solid black. The undiluted sample is shown in light shading; for these values, the log scale along the left Y‐axis. A minimum of three values for each sample were acquired and triplicate values all agreed to within 10%.

An objective of this study was to use the library screening approach to identify candidate peroxidases for the oxidative degradation of fluorinated organic substrates; therefore, the same panel of crude samples was evaluated in the conversion of the probe substrate (4FP) into the primary oxidized benzoquinone product, as shown in Figure 2. An HPLC assay was developed to monitor this reaction. The benzoquinone product had a retention time of 1.9 min and absorbed optimally at 254 nm. A standard curve was generated for the quinone product, and activities were assessed for the undiluted, 1:10, and 1:100, dilution samples. Many of the samples that showed measurable activity in the guaiacol assay had detectable activity with fluorophenol as a substrate as well (Figure 3). As with the guaiacol assay results, PKS and BTS, along with HRP, exhibited the highest overall activity of the samples examined. Using the 1:100 dilution, the PKS and BNS were about 10x more active than the HRP sample.

**FIGURE 4:**
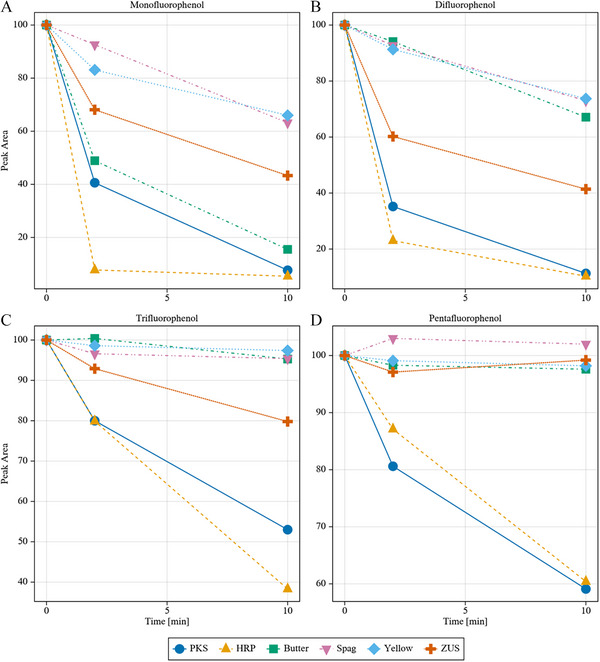
Comparative rates of degradation of (A) 4FP, (B) DFP, (C) TFP, and (D) PFP by each of the peroxidase samples examined. Each point represents a minimum of two independent trials, and error bars are within the size of the symbols used. 4FP indicates 4‐fluorophenol; DFP, 3,4‐difluorophenol; PFP, 2,3,4,5,6‐pentafluorophenol; TFP, 3,4,5‐trifluorophenol

Although PKS and BNS showed the highest overall activity in the crude extracts, this did not necessarily mean that these peroxidases had higher intrinsic activity, rather it may simply reflect a much higher abundance of peroxidase in the original source. Based on the data acquired using 4FP (Figure 3), it was apparent that samples from the skin of the fruit from the *Cucurbita* genus were most promising in terms of total peroxidase content and de‐fluorinating activity, so six samples from this family were selected and examined, alongside of HRP, for their ability to degrade increasingly fluorinated phenolic substrates including DFP, TFP, and PFP. For these experiments, all peroxidase samples were normalized based on the heme content of the crude samples. It was assumed that the amount of extractable heme from each aqueous plant extract could approximate the total peroxidase enzyme in the sample. An HPLC method was developed in which to quantify total heme in a sample using a standard curve approach where known amounts of commercial HRP were used as a standard. This method produced a linear correlation between concentration and peak area over a range of HRP concentrations from 0.2 to 25 µM. The total heme concentrations for crude extracts from BNS, HRP, PKS, ZUS, YS, SPG, and ASP were determined. Figure 4 illustrates the time‐dependent degradation of 1.0 mM solutions of PFP, TFP, DFP, and 4FP in a reaction with 0.06 µM of each of the squash skin peroxidase samples, along with HRP. Quantitative studies involving the HRP reaction with 4FP showed that quinone formation accounted for roughly 85% of the degraded substrate. It should be noted that the activity of ASP was very low for each of the substrates; therefore, the data for ASP are not shown in the figure. Based on these data, several general trends were observed.

The HRP and PKS samples maintained the highest rates of degradation for each substrate, with HRP being the most active on the mono‐ and di‐fluoro compounds, while PKS showed the highest activity with the highly fluorinated PFP. In addition, the rate of degradation by HRP decreased significantly with increasing fluorination. For example, for the 4FP approximately 90% of the substrate was degraded within 2 min, while with the PFP, only 12% was degraded in the same amount of time. In contrast, metabolism by PKS went from 60% of the 4FP down to 20% with PFP. Another observation of note was the behavior of ZUS, which degraded 50% of the 4FP over 2 min, but did not degrade any of the polyfluorinated compounds significantly under the same conditions. The combined observation, that, while HRP appeared to be much more active in the normalized experiments when using the mono‐ and di‐substituted phenols, the PKS displayed a higher rate of degradation of PFP than HRP, is intriguing.

To explain the aforementioned observations, the electron‐withdrawing effects of the fluorine or steric effects due to the increased size of the fluorine atoms were considered. Due to the electronegativity of the fluorine atoms, increasing the number of these groups on the aromatic ring has several predictable effects on the properties of the phenol that could potentially contribute to the observed effects. The effect on p*K*a of the phenol by electron‐withdrawing groups is well established. For the substrates used in this study, the p*K*a values for PFP, TFP, DFP, and 4FP are 5.5, 8.0, 8.9, and 9.9, respectively. Increasing the number of fluorine atoms, therefore, systematically increases the ratio of ionic‐to‐neutral form of the phenol. At pH 7, PFP would be predominantly ionic while all the other substrates would be in their neutral form, thus the PKS active site may be more accommodating of the ionic form in binding compared with the HRP sample. Additionally, the withdrawing effects of the fluorine would be expected to make the oxidation of the phenol thermodynamically more difficult with increasing fluorination, thus the differential abilities of the peroxidases to oxidize the substrates may have its origins in the reduction potential of the enzymes.

**FIGURE 5:**
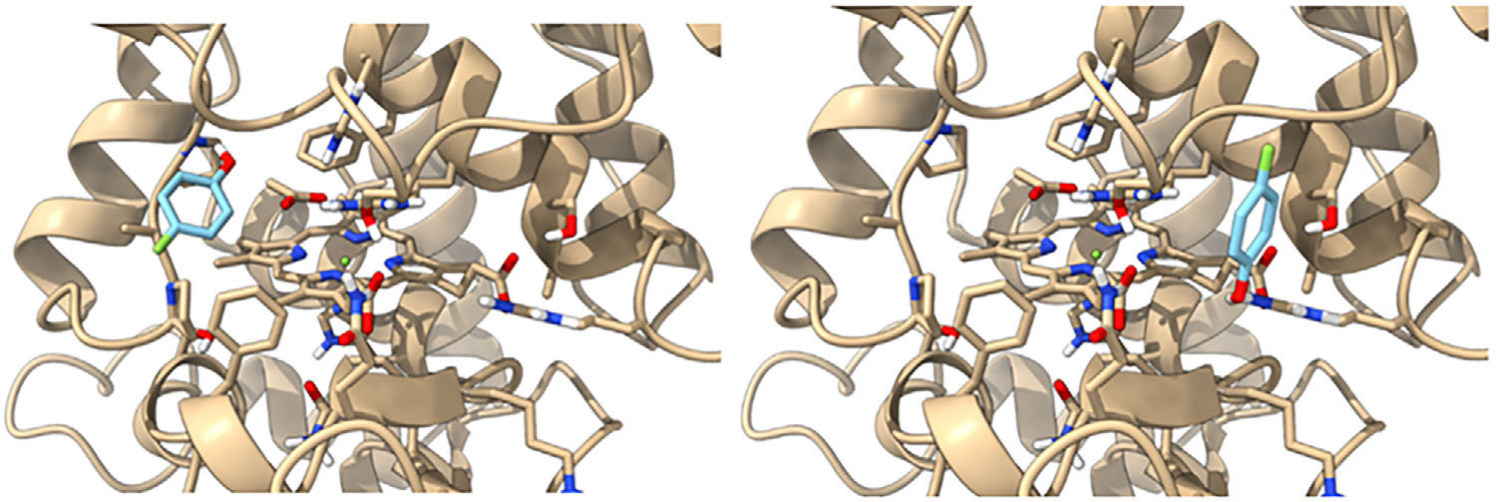
(Left) docking of the substrate 4‐FP at the δ‐site of the HRP enzyme. (Right) structure of HRP with 4FP docked at the γ‐site. Both structures were generated using the crystal structure for HRP (1H5A) and the AutoDock vina. 4FP indicates 4‐fluorophenol; HRP, horseradish root.

Alternatively, steric effects of the F atoms could also play a role in the observed effects on catalysis. To assess steric effects on binding, docking studies were carried out in AutoDock Vina to determine optimum binding distances and orientations for each substrate at the heme edge of HRP, along with the corresponding binding energies for each. Figure 5 shows the 4FP substrate docked to the surface of the HRP enzyme in either of two potential binding sites (either δ‐ or the γ‐heme edge). Prior studies have indicated substrate docking at the δ‐edge is the preferred site for catalysis in HRP [35, 36]; however, related proteins—such as ascorbate peroxidase—are believed to utilize the γ‐edge for catalysis [37]. It is also possible that in HRP the site of catalysis is substrate dependent, which is why both sites were modeled in the current study. Similar structures were generated for each of the remaining substrates used in the study.

For each of the substrates, the protonated form was docked, although at the pH used in the kinetic experiments, the PFP equilibrium likely favored the anionic form, thus an additional docking was carried out with the anionic form. A comparison of the minimum binding energies related to all four substrates docked at the δ‐ or γ‐site (Table 2) indicated only minor differences for the mono, di, and tri‐fluorophenol. With the PFP, the binding was actually more favorable at the g‐site, although interactions at both sites were much weaker than with the less substituted compounds. Interestingly, in the anionic form, PFP did not appear to bind to the δ‐site at all, only to the γ‐site, and with less favorability than the protonated form (data not shown). These observations are consistent with a model in which the higher observed relative activity of the PKS with respect to HRP in the metabolism of increasingly fluorinated phenols may be related to weaker binding interactions at HRP, coupled to the lower p*K*a, producing an increased proportion of the phenolate anion, which binds very poorly to HRP.

**TABLE 2:** Binding energies and phenol‐heme distances for each of the substrates examined.

Molecule	δ‐Energy (kcal mol^−1^)	δ‐Distance (Å)	ɣ‐Energy (kcal mol^−1^)	ɣ‐Distance (Å)
4FP	−3.008	4.542	−2.869	8.866
DFP	−3.474	4.561	−2.71	10.199
TFP	−2.866	5.374	−2.586	12.364
PFP	−0.75	6.275	−1.147	9.22

*Note:* The distances were measured from the phenolic oxygen to the corresponding meso‐carbon position on the heme.

Abbreviations: 4FP, 4‐fluorophenol; DFP, 3,4‐difluorophenol; PFP, 2,3,4,5,6‐pentafluorophenol; TFP, 3,4,5‐trifluorophenol.

Another interesting feature of these docking studies was that the distance between the phenolic oxygen in the substrate and the δ‐meso carbon of the heme increased with increasing fluorination. This trend correlated well with the degradation rates, consistent with observations by Khopde and Priyadarsini, who also concluded that distance has a profound impact on the outer sphere electron transfer from substrate to heme in HRP [38]. These distances are also shown in Table 2. Alternatively, the electron withdrawing properties of F will make electron removal more difficult as more F atoms are added to the ring. The consequent effect on the oxidation potential of the phenol may establish a thermodynamic barrier to electron transfer and oxidation of the substrate and could also explain the observed trends. For example, the BNS sample was very high in its relative activity with 4FP, whereas no measurable activity was observed with either TFP or PFP. Assuming a classical peroxidase mechanism is involved for all reactions, a lower reduction potential for the compound I or II associated with BNS compared with HRP or PKS could also account for the inability to oxidize the more highly fluorinated substrates. A more detailed kinetic characterization of the peroxidases would provide the necessary information for distinguishing between binding versus catalysis being the underlying feature driving the observed trends, which is certainly recommended for future studies.

An additional feature of the data was that in each case the kinetics were distinctly non‐linear, suggestive of some type of inhibition that is reaction dependent. Fluoride ions have been shown to inhibit many heme‐containing enzymes, including peroxidases; however, prior studies have shown a lack of effect for fluoride ions on HRP up to 0.25 mM concentrations [39]. It is possible that different peroxidases have different sensitivities to fluoride ion, and thus additional studies aimed at exploring the fluoride sensitivity of a diverse group of peroxidases may be warranted. Alternatively, based on subsequent studies by Pirzad et al., a fluorophenol radical is the likely source of the non‐linear kinetics observed [30].

Several recent articles have described methods for the degradation of fluorinated phenolic compounds and have highlighted the importance of this process [40, 41]. The peroxidases described here are readily available from agricultural products that are very low cost to harvest and relatively simple to process. The PKS, in particular, consistently gave the highest peroxidase yield per gram of crude material. For example, on average, 1.0 g of PKS produced about 16x more peroxidase (based on heme concentration) than the equivalent amount of HRP. In other words, 1 oz of Pumpkin skin contains as much peroxidase as a lb of Horseradish root. Clearly, the PKS provided the highest yield of activity per gram of tissue, making it an excellent candidate for continuing studies for the discovery of effective low‐cost catalysts for the degradation of fluorinated phenolic compounds. Furthermore, perhaps the addition of BSA could further enhance the overall efficiency of the process by effectively inhibiting or delaying inactivation of the PKS enzymes, but this must be validated experimentally. Finally, the implementation of low‐cost peroxidases, such as PKS in combination with photocatalytic enhanced surface area structures such as those reported in [42, 43, 44], could lead to the design of low‐cost bioreactors capable of efficiently destroying PFAS contaminants.

## 4 Concluding Remarks

Polyfluorinated aromatic compounds (PFACs) are a subset of PFAS that have been used in the pharmaceutical industries for many years. The aromatic nature of these compounds makes them easier targets for chemical degradation, thus they represent a good initial target for the development of model enzymatic systems for degradation, which could be eventually translated to many other types of PFAS.

In this study, we demonstrate the utility of a “library” approach in evaluating the catalytic properties of a large number of plant peroxidase enzymes, specifically as it relates to the degradation of fluorinated aromatic compounds. We have identified several peroxidase enzymes from the *Cucurbita* family that are produced in high abundance and possess promising catalytic capabilities. For example, PKS, the peroxidase from the skin of pumpkin (*Cucurbita* maxima), degraded mono‐, di‐, tri‐, and penta‐fluorophenols with high catalytic activity. Although a very profound reduction in activity was observed for HRP with increasing fluorination of the phenolic substrate, PKS showed only modest reductions in activity. Docking studies using the known crystal structure of HRP suggested that the active site of HRP does not accommodate the steric bulk of the additional fluorines well, causing the substrate to dock further from the catalytic heme, thus slowing the rate of catalysis. We suggest that the PKS active site may be larger providing closer access to the perfluorinated substrate and thus retains higher activity relative to the HRP enzyme. The high catalytic activity combined with the very high yield of peroxidase per gram of tissue makes the PKS enzyme an excellent candidate for development of environmentally friendly biocatalytic strategies for the degradation of fluorinated aromatics.

## Conflicts of Interest

The authors have declared no conflicts of interest.

## Data Availability

Supporting data is available from the corresponding authors upon reasonable request.
